# How to develop the subspecialty of pediatric urology?

**DOI:** 10.4103/0970-1591.36712

**Published:** 2007

**Authors:** Paddy Dewan

**Affiliations:** Wee Kids Urology Research Unit, Melbourne, Victoria, Australia

***What an interesting question!***

The short answer is to seek the input of all those with an interest in the care of the urinary tract in the fetus, the child and the adolescent and then for those groups to work collaboratively with the surgeons who take on the task of subspecializing in Pediatric Urology. Implementation of the development of the subspecialty has to include infrastructure support, include training programs, certification, accreditation and research funding.

However, the solution is not that simple, as one of the features of the medical profession is competition between practitioners and competition between specialties. We care about our patients, we work hard for them and most medical professionals work tirelessly for either colleges, medical associations, journals or medical schools, while others put their effort into research. There are those who contribute toward many facets of their surgical profession.

A fact of medical life is that each of those component parts involves both collaboration and competition with others, both for funds and patient access. The competition for patients who generate income and more so, for interesting cases on which we build a career, is not at all surprising. After all, medicine is a human pursuit. Good leadership in institutions, colleges, universities and hospitals keeps the lid on the competition between groups and individuals, facilitating a commonality of purpose through collaboration.

Pediatric urology is not new, but is a young subspecialty, thus there are many who feel ownership of the “patch” and the patients, including Pediatricians. Managing these tensions is important to ensure that progress is made in the understanding of diseases that affect the pediatric urinary tract. Thus far, these tensions between groups have not been well managed, leaving us with still much to learn about the diseases we treat as pediatric urologists. Whenever a new area of expertise unfolds there is always resistance to its development and argument against change - yet another human endeavor default position.

In Australia there is no formal recognition of the specialty of Pediatric Urology, which is the case in most countries. The answer to the question of who looks after children with anomalies of the urinary tract and related diseases, tends to develop in an *ad hoc* manner, depending on the personalities and their level of influence in each center. The state of the turf war between the Urologists and Pediatric Surgeons depends on the country and the hospital with the predominance of one or other group being on the basis of politics rather than quality considerations. A statement that could be construed as insulting, which it is not - the politically strong group may also be that which is best equipped to provide the higher standard, but we have no ready measurement to make that judgment.

Importantly, it should be realized that there is a wide range of specialties that provide care for children with conditions that a Pediatric Urologist might treat, including Pediatricians, Obstetricians, General Surgeons, Pediatric Surgeons and Urologists. Expertise is also provided by Radiologists, Pediatric Radiologists, Nuclear Medicine Physicians and Ultrasonologists, all with a valid claim to having knowledge of the disease of the diseases they investigate. A practitioner in Pediatric Urology requires the assistance of staff with not only the skill to interpret the investigations, but with the ability to deal with children in a manner that maximizes the potential to acquire the information needed to correctly make a diagnosis.

Each of these different groups has different skills in diagnosis and management: a medico with subspecialty training in diseases of the adult genitourinary tract will have a greater understanding and experience with tumors, endoscopy, stone disease and strictures. A person with training in Pediatric Surgery will have had more training and experience in managing the resuscitation of neonates, prenatal diagnosis, congenital anomalies and the related pathology of anorectal anomalies. There is also a difference in the appropriateness of open surgery with children and adults.

The question should not be who is doing the surgery, nor what is their training, but how good is the care, how good are their technical skills and is the care being provided in an environment that is conducive to a good outcome. We do not have good systems to judge how good a surgeon the Pediatric Urologist is and we do not know, for certain, what is the correct treatment, of an individual patient, in many of the conditions we manage. For hypospadias, for example, we can look at the stricture and fistula rate, to assess outcome but with conditions such as obstruction of the urinary tract and vesicoureteric reflux there is such a breadth of opinion that it is virtually impossible to judge one surgeon against the next. To develop any specialty we need to develop performance criteria that relate to the outcome of the disease, not just of operations.

By exploring specific diseases in detail, we see the difficulties and some of the solutions, to how we should develop the specialty of Pediatric Urology. Let us look at hypospadias, pelviureteric junction obstruction and congenital urethral obstruction as examples. Superficially, one can become a hypospadiologist, pyeloplastician or surgeon trained to manage boys with urethral obstruction by working in a center that has a sufficient case-load of bent penises and blocked kidneys or by training as a Urologist or Pediatric Surgeon and then having further training in a center that deals with these cases in sufficient numbers to enhance the skills of the Pediatric Urology aspirant.

However, hypospadias is a disease that requires knowledge of a range of techniques and surgical steps including chordee release, staged procedures, dorsal plication, ventral corporal grafting, penile dismemberment procedures, as well as urethral replacement. Otherwise the surgeon is a Urologist or Pediatric Surgeon with an interest in Pediatric Urology, not a Pediatric Urologist. This knowledge can be partly gained on the job, but the subspecialist status also requires the time and commitment to review the literature and participate in meetings where these issues are being discussed: a theme that is common to all aspects of all subspecialties.

Turning to the second illustrative disease of pelviureteric junction obstruction. In recent years, in Papua New Guinea, this condition was treated by nephrectomy, but now, pyeloplasty is performed on late presenting patients by surgeons who have been trained by a Pediatric Urologist, but there is no Pediatric Urologist in Papua New Guinea - there is not yet the need for the subspecialty. In contrast, in Australia and India, and many countries in the world, a large proportion of children requiring an operation for an obstructed kidney will have had a diagnosis of hydronephrosis prior to birth. Therefore, there is a raft of expanding knowledge that should be held by the surgeon treating upper renal tract obstruction in a majority of countries. How well a surgeon performs a pyeloplasty and the obstruction rate, is hardly a judge of their skill as a Pediatric Urologist. The candidate must have more than cursory experience in prenatal ultrasound, post natal ultrasound, nuclear medicine, pediatric radiology and be aware of the range of rarer anatomical and diagnostic variations in order to ensure that the outcome for all patients is maximized. Importantly, the Pediatric Urology fraternity should provide the leadership that promotes and conducts research that will take us beyond the current confusion that predominates in the debate on both obstruction and reflux in the pediatric urinary tract.

Posterior urethral obstruction is another conditioning, in the field of the Pediatric Urologist, which demands a wide knowledge, if the practitioner is to deliver the best care to the patients: these boys are often detected prenatally and may be considered for prenatal intervention. The Pediatric Urologist should be able to contribute to the discussion about the nature and timing of any prenatal intervention and should be aware of the options being explored at the cutting edge of knowledge, such as fetal ablation of the obstruction: involvement in that discussion should not even commence if the person claiming to be a Pediatric Urologist is not aware of recent considerations about the anatomy.

Once a boy is born with posterior urethral obstruction, the center in which he is treated should be able to provide the skills of a neonatal intensive care unit, Pediatric Radiologists and Pediatricians able to deal with lung and renal resuscitation. The Pediatric Urologist, through his training, has to be familiar with the management of post obstructive diuresis in these children, the options for drainage, including the use of various diversion techniques. Thereafter, the surgeon will need to have ongoing input into decisions on bladder management, should be able to consider modern options of bladder augmentation and then be ready and able to counsel the family on the role and timing of transplantation.

Surgeons can participate in a limited range of cases that may fall in the specialty of Pediatric Urology, such as circumcision orchidopexy, testicular torsion, minor hypospadias and if trained in Urology, stone disease and strictures, but rare conditions such as cloacal anomalies, major hypospadias and bladder augmentation should be undertaken by a limited number of well-trained people, who have sufficient ongoing experience and the time to keep abreast of research and other developments.

Establishing the specialty should involve identifying the case-load of the major Pediatric Urology cases, estimation of the manpower to both provide the patient service and enable sufficient ongoing experience for each of the members of the team for these major cases. Pediatric Urological services should not be modeled on the number of minor cases that are likely to be dealt with by the incumbents. The service for the other, more common anomalies, would be appropriately be provided by those who have been trained to deal with that part of the case-load. The more common anomalies should be shared around. The Pediatric Urology Unit needs to be provided with the infrastructure that will support the clinical needs of the service and financial support for research.

If surgeons do not manage the manpower of their specialties, we end up as technicians who are told when to operate, as the physicians, who are often the first point of referral of Pediatric Urology cases, tend to refer according to how they want the cases managed, rather than allowing for the surgical expertise to predominate, as control of the patients are then less in their hands. This difficulty with loss of control is another of the human behavior realities that impacts the practice of Pediatric Urology, which needs to be managed.

Those wishing to become a Pediatric Urologist should be supported by both the Urological and Pediatric Surgical community to obtain the skills for them to become the innovators in care of the Pediatric urinary tract, not surgeons who create a monopoly. However, we do need to ensure that we promote the worth of the Urological perspective in the interpretation of research of the urinary tract in children.

In Australia, Urologists and Pediatric Surgeons have come together and established a Pediatric Urology Club. This *ad hoc* group goes part way to establishing an organization that can oversee the development of the subspecialty, but the group has no constitution and no rules of engagement. So, while it provides for a meeting of minds from both the specialties through which one can gain the ability to deal with Pediatric Urology patients, it does not set standards for diagnosis, management or research. Importantly, it does not enable subspecialists to be seen as the leaders of the development of care of the Pediatric Urology disease, but considers them to be one of a group of opinion holders. Nevertheless, the Pediatric Urology Club is a step in the right direction; it facilitates collaboration.

The training is life-long, but the training program needs to be finite and, importantly, not everyone can become an expert in all aspects of Pediatric Urology. Some will be very good at one or other aspect of the specialty; our task in setting up the specialty should include providing standards that are, unlike most in surgical training, a measure of the technical and decision-making skills, rather than the usual exit exam, which is a measure of participation in the academic process.

The curriculum should include embryology, tumors, congenital renal tract anomalies, knowledge of associated anomalies, management of the neonate, infant and child, Radiology of the fetal and Pediatric urinary tract, operative, percutaneous, extracorporeal, endoscopic and laparoscopic surgery, transplantation, urinary tract reconstruction, urodynamics and incontinence, stone disease and stricture management.

In summary, Pediatric Urology should be developed through a collaboration of those specialties interested in the care of the Pediatric Urinary tract setting a curriculum, establishing training posts and working to develop the infrastructure to support the graduates. The trainees should be exposed to education from Urologists and Pediatric Surgeons who have an interest in the specialty and in centers with subspecialist Pediatric Urologists. Their assessment should be of skills rather than just knowledge and their contribution to the specialty should be assessed in terms of measures of quality, rather than simply throughput (although throughput of major cases should be one of the *de facto* measures of skill maintenance). Also, research output should be one of the performance criteria.

I realize I am stargazing, but without dreams there is no advance.

